# Mitochondrial Ca^2+^ Overload Leads to Mitochondrial Oxidative Stress and Delayed Meiotic Resumption in Mouse Oocytes

**DOI:** 10.3389/fcell.2020.580876

**Published:** 2020-12-15

**Authors:** Luyao Zhang, Zichuan Wang, Tengfei Lu, Lin Meng, Yan Luo, Xiangwei Fu, Yunpeng Hou

**Affiliations:** ^1^State Key Laboratory of Agrobiotechnology, College of Biological Sciences, China Agricultural University, Beijing, China; ^2^Key Laboratory of Animal Genetics, Breeding and Reproduction, Ministry of Agriculture and National Engineering Laboratory for Animal Breeding, College of Animal Science and Technology, China Agricultural University, Beijing, China

**Keywords:** mitochondria, calcium overload, oocytes, meiosis, obesity

## Abstract

Overweight or obese women seeking pregnancy is becoming increasingly common. Human maternal obesity gives rise to detrimental effects during reproduction. Emerging evidence has shown that these abnormities are likely attributed to oocyte quality. Oxidative stress induces poor oocyte conditions, but whether mitochondrial calcium homeostasis plays a key role in oocyte status remains unresolved. Here, we established a mitochondrial Ca^2+^ overload model in mouse oocytes. Knockdown gatekeepers of the mitochondrial Ca^2+^ uniporters Micu1 and Micu2 as well as the mitochondrial sodium calcium exchanger NCLX in oocytes both increased oocytes mitochondrial Ca^2+^ concentration. The overload of mitochondria Ca^2+^ in oocytes impaired mitochondrial function, leaded to oxidative stress, and changed protein kinase A (PKA) signaling associated gene expression as well as delayed meiotic resumption. Using this model, we aimed to determine the mechanism of delayed meiosis caused by mitochondrial Ca^2+^ overload, and whether oocyte-specific inhibition of mitochondrial Ca^2+^ influx could improve the reproductive abnormalities seen within obesity. Germinal vesicle breakdown stage (GVBD) and extrusion of first polar body (PB1) are two indicators of meiosis maturation. As expected, the percentage of oocytes that successfully progress to the germinal vesicle breakdown stage and extrude the first polar body during *in vitro* culture was increased significantly, and the expression of PKA signaling genes and mitochondrial function recovered after appropriate mitochondrial Ca^2+^ regulation. Additionally, some indicators of mitochondrial performance—such as adenosine triphosphate (ATP) and reactive oxygen species (ROS) levels and mitochondrial membrane potential—recovered to normal. These results suggest that the regulation of mitochondrial Ca^2+^ uptake in mouse oocytes has a significant role during oocyte maturation as well as PKA signaling and that proper mitochondrial Ca^2+^ reductions in obese oocytes can recover mitochondrial performance and improve obesity-associated oocyte quality.

## Introduction

Obesity or diabetes induced by high sugar and fat diets are occurring at epidemic rates worldwide (Pan et al., 1997). Many researches have suggested that obesity has detrimental effects on female reproduction—obese women take longer to conceive, even if ovulation cycle regular, and have a higher risk of miscarriage, preeclampsia, and congenital defects in offspring (Pan et al., 1997; Krishnamoorthy et al., 2006; Grindler and Moley, 2013). Previous studies have also shown that type I diabetes can decrease mammalian oocyte quality severely (Pan et al., 1997; Grindler and Moley, 2013), and oocyte maturation and ovulation rates for type I diabetics are considerably lower than those of healthy controls (Wang et al., 2012). These phenomena suggest that defects in oocyte quality contribute to damaged fecundity caused by obesity or diabetes.

Mitochondria are the most abundant organelles in mammalian oocytes and early embryos (Dumollard et al., 2003). They are the main cellular energy producers maintaining the reproductive process. Emerging evidences have suggested that mitochondria provide cellular energy critical for oocyte meiosis progression (Van Blerkom, 2011; Gibson et al., 2005). Mitochondrial dysfunction in oocytes from obese or diabetic mice is associated with poor fertilization rates and abnormal embryo development (Wang et al., 2009). As mitochondria are maternally inherited (Wang et al., 2009), no new mitochondrial could be produced until early preimplantation embryo stage. Research has focused on enhancing mitochondrial functionality to improve oocytes quality. Studies on improving obese mouse oocyte quality have reported that transplantation of mitochondria from healthy mouse oocytes into obese ones can increase oocyte quality and enhance mitochondrial functionality in their offspring (Kristensen et al., 2017). The treatment of some antioxidant drugs—such as glutathione, melatonin, and resveratrol—can ameliorate oocyte maturation by recovering damaged mitochondrial performance (Boots et al., 2016; Han et al., 2017). These results indicate that the activity of mitochondria plays a key role in maintaining oocyte quality.

Calcium (Ca^2+^) is a second messenger that mediates many physiological processes—such as differentiation, apoptosis, and oxidative stress. Calcium signaling homeostasis plays an important role in maintaining cellular processes. Evidence suggests that Ca^2+^ dysregulation can give rise to neurodegenerative diseases through oxygenated stress damage (Penna et al., 2018). Previous studies have shown that both endoplasmic reticulum and mitochondria were primary Ca^2+^ stores which maintained cellular calcium homeostasis (Wang et al., 2017). Mitochondrial Ca^2+^ homeostasis controls several biological processes in the cell (Paillard et al., 2017), and the accumulation of mitochondrial Ca^2+^ may alter mitochondrial morphology, redox state, and ATP production (Han et al., 2017). Previous research showed that oocytes from obese mice exhibit higher mitochondrial Ca^2+^ levels (Zhao et al., 2017), which is consistent with our study in diabetes and aged mice (unpublished data). These oocytes have shown impairment of meiotic maturation and exhibited many of the characteristics recently observed in the obese, diabetic, or aged mice (Ben-Meir et al., 2015; Hou et al., 2016). However, from current studies, it is not clear whether mitochondrial Ca^2+^ overload is attributable to problems that arise in the obesity, diabetes, or aged mice oocytes. The role of mitochondrial Ca^2+^ overload in regulating oocyte quality and meiosis maturation is still unknown.

More recently, it has been shown that Ca^2+^ can also play a central role in triggering some controlled pathway of mitochondrial function. As a result, oocyte mitochondria produce less ATP and more reactive oxygen species (ROS) that can damage multiple components of the cell including DNA, RNA, proteins, and lipids and, thereby, perturb diverse biological processes—such as cell metabolism, apoptosis, and aging. ATP is an important indicator of mitochondrial function, and ATP deficiency resulting from mitochondrial dysfunction may be a common denominator for an array developmental defects (Kahn et al., 2005; May-Panloup et al., 2007).

Previous research showed that ATP concentration can affect the AMP-activated protein kinase (PKA) signaling pathway and has a relationship with PKA phosphorylation in somatic cells (Kahn et al., 2005). The PKA signaling pathway is sensitive to the AMP/ATP ratio in mouse oocytes (Bertoldo et al., 2015). PKA has been shown to improve resumption of oocyte meiosis in mice (Chen and Downs, 2008; Reverchon et al., 2013), however, the interactions between mitochondrial Ca^2+^ and PKA signal during oocyte maturation remain unclear.

In addition, the mitochondrial Ca^2+^ uniporter (MCU) mediates the calcium-dependent physiological stimulation of oxidative reactions to avoid mitochondrial Ca^2+^ overload and cell death (Patron et al., 2014). As an indispensable component of the MCU complex, the calcium-sensing protein Micu1 acts as a gatekeeper to avoid mitochondrial Ca^2+^ overload (Paillard et al., 2018). A previous study showed that Micu1 deletion increases mitochondrial Ca^2+^ concentration, and that Micu2 has a similar effect in regulating mitochondrial Ca^2+^ homeostasis in HeLa cell (Csordas et al., 2013). Extruding Ca^2+^ from the mitochondrial matrix occurs primarily through a mitochondrial Na^+^/Ca^2+^ exchanger (NCLX) (Luongo et al., 2017). Recent studies have indicated that damaged NCLX activity leads to mitochondrial Ca^2+^ overload and defects in mitochondrial function (Luongo et al., 2017).

In this study, we established two mouse oocytes models: (1) knockdown of Micu1 or Micu2 and (2) knockdown of NCLX. The aim of this study was to determine whether mitochondrial calcium homeostasis plays an important role in meiotic resumption and whether impaired oocyte mitochondrial function can be improved by decreasing mitochondrial Ca^2+^ concentration.

## Materials and Methods

### Ethics Statement

All chemicals were purchased from Sigma Chemical Co (St Louis, MO, USA) unless otherwise stated. All animal manipulations were performed according to the guidelines of the Animal Care and Use Committee. The present study was approved by the Institutional Animal Care and Use Committee of China Agricultural University (AW01040202-1).

### Generation of Obese Mice

Obese mice and oocyte harvesting were carried out as previous reported (Zhao et al., 2017). CD-1® (ICR) female mice (3 week-old) were purchased from the Beijing Vital River Experimental Animals Centre (Beijing, China) and housed under 12 h light: dark cycles at a temperature of 23 ± 2°C for all experiments. The mice were randomly divided into two groups (five per cage): one group was fed a control diet (CD) and the other was fed a high fat diet (HFD) for 12 weeks with free access to food and water. After obesity had been established, mice from the two groups were weighed.

### Oocyte Collection and Culture

Before all experiments, the mice were treated with 5 IU of pregnant mare serum gonadotropin (PMSG) for 46–48 h and then sacrificed by cervical dislocation. Germinal vesicle (GV) stage oocytes were collected for subsequent experiments. All procedures were performed under the Institutional Animal Care and Use Committee of China Agricultural University (AW01040202-1).

Oocytes were collected from 4 to 6 week-old ICR mice. To obtain GV-stage oocytes, females were primed with 5 IU of pregnant mare serum gonadotropin and euthanized after 46 h. By puncturing the fully grown follicles, GV-stage oocytes were released from the ovaries into pre-warmed M2 medium supplemented with 2.5 μM milrinone. After specific treatments, oocytes were washed thoroughly and cultured in M16 medium, undergoing GV and MII stages.

### siRNA Microinjection

Small interfering RNAs (siRNA) for Micu1 (sequence: AGCCUUAUCCUGAGGACAATTU UGUCCUCAGGAUAAGGCUTT), Micu2 (sequence: CCUCUUCUCAGUCAUGUUUTTAAACAUGACUGAGAAGAGGTT), NCLX (sequence: CCUUCUUGCCACGUCUAATTUUAGACGUGGCAAAGAAGGTT), MCU (sequence: CCAAAGAGACCUAATTUUAGGAGGUCUCUCUUUGGTT) (GenePharma, Shanghai, China), or siRNA control were microinjected (5 μM) into the cytoplasm of fully grown GV oocytes with an Eppendorf microinjection instrument (Hamburg, Germany) and completed within 30 min. Oocytes were arrested in M16 supplemented (Sigma-Aldrich, St. Louis, MO, USA) with 2.5 μM milrinone for 20 h to block mRNA translation. After 20 h, the oocytes were cleaned thoroughly to resume meiosis.

### Measuring Mitochondrial Ca^2+^ ([Calcium]m)

[Calcium] m levels were assessed using Rhod-2AM (Invitrogen/Molecular Probes, Carlsbad, CA, U.S.) according to a previous procedure (Zhao et al., 2017). First, zona pellucida was enzymatically removed by 0.5% pronase 37°C for 5 min. The oocytes were then processed in maturation medium with 5 μM Rhod-2AM for 30 min, washed three times by DPBS, and incubated without Rhod-2AM at 37°C and 5% CO_2_ for 30 min. Subsequently, they were analyzed using a confocal laser scanning microscope (Nikon A1R, Tokyo, Japan) and quantitatively processed using NIS-Elements AR (Nikon Instruments, Tokyo, Japan).

### Mitochondrial Reactive Dye Mito-Tracker (Green)

Mitochondrial distribution was determined using mitochondrial reactive dye Mito-tracker (Green) (Beyotime Institute of Biotechnology, China). The oocytes were then processed in maturation medium with 5 μM Mito-tracker (Green) for 20 min, washed three times by DPBS. Subsequently, they were analyzed using a confocal laser scanning microscope (Nikon A1R, Tokyo, Japan) and quantitatively processed using NIS-Elements AR (Nikon Instruments, Tokyo, Japan).

### Measuring Cytosolic Ca^2+^ ([Calcium]i)

Cytosolic Ca^2+^ levels were assessed using Flou-3 AM (Invitrogen/Molecular Probes, Carlsbad, CA, U.S.). First, zona pellucida was enzymatically removed by 0.5% pronase 37°C for 5 min. The oocytes were then processed in maturation medium with 5 μM Flou-3 AM for 40 min, washed three times by DPBS. Subsequently, they were analyzed using a confocal laser scanning microscope (Nikon A1R, Tokyo, Japan) and quantitatively processed using NIS-Elements AR (Nikon Instruments, Tokyo, Japan).

### Quantification of Mitochondrial Membrane Potential by JC-1 Staining

To measure mitochondrial membrane potential (Δϕm), oocytes were incubated with JC-1 using a mitochondrial membrane potential assay kit (Beyotime Institute of Biotechnology, China). Oocytes were exposed to 10 μM JC-1 in 100 μM working solution at 37.0°C in 5% CO_2_ for 20 min, after which they were washed with washing buffer to remove surface fluorescence and observed using a fluorescence microscope (Olympus IX73). Δϕm was calculated as the ratio of red fluorescence corresponding to activated mitochondria (J-aggregates) to the green fluorescence corresponding to less activated mitochondria (J-monomers).

### ROS Content Assay

The average ROS content in each oocyte was determined by using an Elisa ROS Assay Kit, store in 4°C,TX20634 (Yingxin lab, China) according to the manufacturer's instructions. Denuded oocytes were mixed with 10 μM of RIPA buffer to a 0.2 mL centrifuge tube and then homogenized by overtaxing until lysis occurred. Then, luminescence activity was measured immediately using luminometer (Power Wave XS2). ROS content of samples was determined from the standard curve(U/mL).

### cAMP Content Assay

The average cAMP content in each oocyte was determined by using a cAMP Activity Assay Kit, store in −20°C,K371-100 (Bio vision, the U.S.) according to the manufacturer's instructions. Denuded oocytes were mixed with 10 μM of RIPA buffer to a 0.2 mL centrifuge tube, and then homogenized by overtaxing until lysis occurred. Then, luminescence activity was measured immediately using luminometer (Power Wave XS2). cAMP content of samples was determined from the standard curve (pmol/l).

### ATP Content Assay

The average ATP content in each oocyte was determined by using an Enhanced ATP Assay Kit, S0027 (Beyotime Institute of Biotechnology, China) according to the manufacturer's instructions. Serial dilutions of ATP standard were prepared before examining, ranging from 0 to 40 pol ATP. Ten denuded oocytes were mixed with 10 μM of lysis buffer to a 0.2 mL centrifuge tube on ice, and then homogenized by overtaxing until lysis occurred. All procedures were operated on ice before measurement. ATP assay buffer were added to 96-well plates and equilibrated for 3–5 min at room temperature. Then, standard solutions and ATP detection diluent were injected into each well. Subsequently, samples were also added into each well and luminescence activity was measured immediately using luminometer (Infinite F200; Tecan). ATP content of samples was determined from the standard curve. The total amount of ATP was divided by the number of oocytes in each sample to obtain the mean content per oocyte (pmol/oocyte).

### Measuring Intracellular ROS Levels

Intracellular ROS levels were measured as described previously (Zhao et al., 2017). Oocytes were incubated in M2 supplemented with 1 mmol/L 2′,7′-dichlorodihydrofluorescein diacetate (H2DCFDA) for measuring ROS for 30 min at 37°C and washed three times. The fluorescence was examined under an epifluorescence microscope with a filter at 460 nm excitation for ROS (DP72, Olympus, and Tokyo, Japan). All data were analyzed using ImageJ software.

### Semi-Quantitative Reverse Transcription PCR (RT-PCR) and Quantitative Real-Time PCR (qRT-PCR)

Total RNA was extracted from 40 collected GV oocytes using a RNeasy micro-RNA isolation kit (Qiagen, Valencia, CA, and U.S.) following the manufacturer instructions. The RNA concentrations were measured using a NanoDrop 2000 Spectrophotometer (Biolab, Scoresby, Victoria, Australia) at wavelength of 260 nm. We wouldn't use the samples for subsequent analyses until their absorbance ratio at 260 nm: 280 nm >1.8.

Reverse transcription was conducted to generate cDNA libraries using a Quantitated Reverse Transcription Kit (Qiagen) according to the manufacturer instructions and we treated the sample with DNaseI before that. QRT-PCR and RT-PCR were performed using an ABI 7500 real-time PCR instrument and a Fast 96-well Thermal Cycler (Applied Biosystems, Foster City, CA, U.S.). The sequences of all primers used are listed in Supplementary Table 1. The relative expression of genes was calculated with the comparative threshold cycle (CT) method as 2^−ΔΔCT^.

### RNA Sequencing

We performed expression profiling on pools of 40 denuded GV-oocytes isolated per group. RNA was isolated using the RNeasy Micro Kit (Qiagen). cDNA was generated and amplified from 1.2 ng with the Nu-Gen ovation RNA-seq System V2 (Part no. 7102; Nu-Gen). 50 ng of the resulting SPIA cDNA was fragmented and sequencing libraries were prepared using Tru-Seq DNA Sample Preparation Kit (low-throughput protocol) (Part no. 15005180 Rev. C; Nu-Gen).

The sequencing data was filtered with SOAP-nuke (v1.5.2) by (1) Removing reads containing sequencing adapter; (2) Removing reads whose low-quality base ratio (base quality ≤5) is more than 20%; (3) Removing reads whose unknown base (“N” base) ratio is more than 5%, afterwards clean reads were obtained and stored in FAST-Q format. The clean reads were mapped to the reference genome using HISAT2 (v2.0.4) Bowtie2 (v2.2.5) was applied to align the clean reads to the reference coding gene set, then expression level of gene was calculated by RSEM (v1.2.12) (https://github.com/deweylab/RSEM). The heatmap was drawn by pheatmap (v1.0.8) according to the gene expression in different samples.

### Differential Expression Analysis

Differentially expressed genes and repeat elements were identified Phyper based on Hypergeometric test, by fitting a three-factor model of the form “KD-control,” “KD-Micu1/2,” and “KD-NCLX.” Only genes with at least 3 reads per million in at least three samples were included in the analysis (11,366 for Ref-Seq annotation and 14,954 for oocyte specific annotation). Differential expression analysis was performed using the DESeq2 (v1.4.5).DESeq2.html with *Q* ≤ 0.05. To take insight to the change of phenotype, GO (http://www.geneontology.org/) and KEGG (https://www.kegg.jp/) enrichment analysis of annotated different expressed gene was performed by Phyper based on Hypergeometric test. The significant levels of terms and pathways were corrected by *Q* value with a rigorous threshold (*Q* ≤ 0.05) by Bonferroni.

### Statistical Analysis

Each experiment was repeated at least three times. A representative image of each experiment is shown. All data were analyzed using *t-*tests followed by the Fisher LSD test and one-way analysis of variance (ANOVA) examined by Duncan's multiple-range test in SPSS software (IBM, Chicago, IL, USA). Data are expressed as the mean ± SEM (ns. represents not significant, *represents *p* < 0.05, **represents *p* < 0.01, ***represents *p* < 0.001).

## Results

### Deletion of Micu1/Micu2 or NCLX Induces Increased Mitochondrial Ca^2+^ Levels in Mouse Oocytes

**Figure 1:**
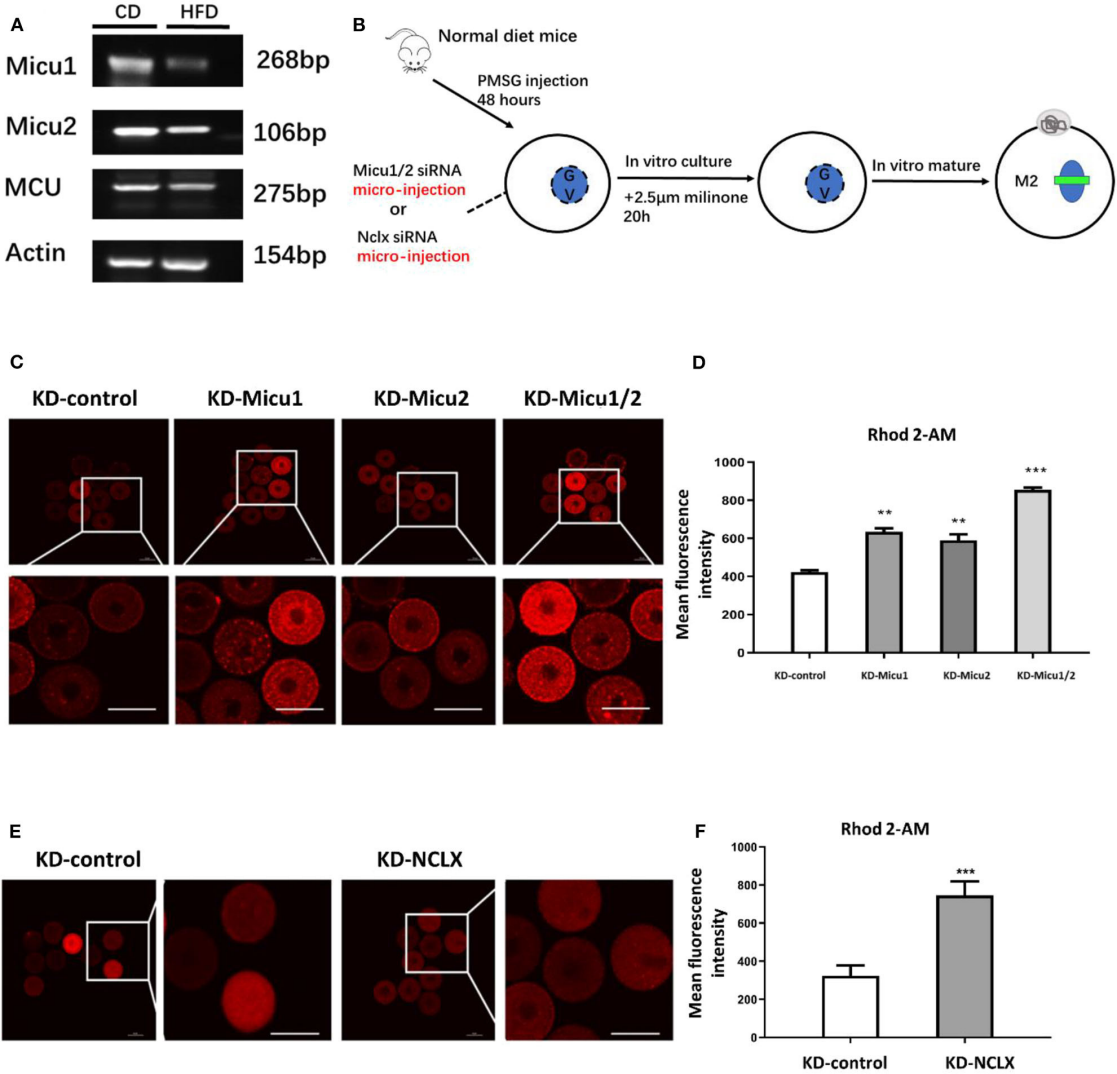
Micu1/Micu2 or NCLX knockdown induces mitochondrial Ca^2+^ overload in mouse oocytes. **(A)** The mRNA levels of Micu1 and Micu2 in obese mouse oocytes. **(B)** The process about establishment of the mouse oocyte calcium overload model by microinjection special siRNA sequence to knockdown expression about Micu1 and Micu2 as well as NCLX in mouse oocytes. **(C)** Representative images of Rhod-2 AM fluorescence (red) in germinal vesicle (GV) stage oocytes treated with KD-control, KD-Micu1, KD-Micu2, and KD-Micu1/2. Scale bar: 50 μm. **(D)** Quantification of the relative levels of mitochondrial Ca^2+^ in KD-control, KD-Micu1, KD-Micu2, and KD-Micu1/2 injected GV-stage oocytes. (*n* = 50 for each group). **(E)** Representative images of Rhod-2 AM fluorescence (red) in GV-stage oocytes from KD-control and KD-NCLX. Scale bar: 50 μm. **(F)** Quantification of the relative levels of mitochondrial Ca^2+^ in GV-stage oocytes from KD-control and KD-NCLX. (*n* = 50 for each group). Student's *t-*test and one-way ANOVA were utilized for statistical analyses. **P < 0.01; ***P < 0.001 vs. control group. Error bars indicate SEM.

Previous research has shown that oocytes from obese mice exhibit higher mitochondrial Ca^2+^ levels, then real-time PCR to analyze the expression of gatekeeper of mitochondrial Ca^2+^ uniporter, Micu1 and Micu2 between control and obese mice. As shown in Figure 1A, the expression of Micu1 and Micu2 in obese oocytes significantly decrease. To generate the mitochondrial overload model in mouse oocytes, we separately targeted Micu1/Micu2 and NCLX for knockdown with siRNA injected into oocytes (Figure 1B, Supplementary Figures 1A–C). Then, we used Rhod-2 AM to analyze mitochondrial Ca^2+^ levels with a confocal laser scanning microscope. To confirm that Rhod-2 AM can be used to show the level of mitochondrial Ca^2+^ specially, we treated the GV oocytes with the mitochondrial reactive dye Mito-tracker (Green) co-stained with Rhod-2 AM. As shown in Supplementary Figure 2, double staining revealed co-localization of these two reactive dyes, declaring that Rhod-2 AM was reliable.

This analysis revealed that Micu1/Micu2 siRNA-injected mouse oocytes (KD-Micu1/2) had a markedly increase in their levels of mitochondrial Ca^2+^ (Figures 1C,D). Knockdown of NCLX with siRNA injection into mouse oocytes (KD-NCLX) also increased mitochondrial Ca^2+^ levels (Figures 1E,F). Additionally, CGP-37157, a specific inhibitor of NCLX was used to validate the siRNA results. Result showed that CGP-37157 was dose-dependent and the effect of 10 μM CGP-37157 on mitochondrial Ca^2+^ of oocytes was equivalent to knockdown of NCLX with siRNA (Supplementary Figure 3A). Moreover, previous studies demonstrated that the increase in intracellular Ca^2+^ directly impacted oocytes maturation (Qi et al., 2015), therefore, we measured intracellular Ca^2+^ levels using Flou-3-AM. As shown in Supplementary Figures 3B,C, quantitative analysis revealed that the relative mean Flou-3 AM intensity in KD-Micu1/2 or KD-NCLX oocytes had no significant difference to the control (Supplementary Figures 3B,C). These observations showed that KD-Micu1/Micu2 or KD-NCLX in mouse oocytes both significantly increased levels of mitochondrial Ca^2+^ while intracellular Ca^2+^ levels were unaffected. Since then, we confirmed that KD-Micu1/Micu2 or KD-NCLX would be an available model to research the potential impact of mitochondrial Ca^2+^ overload on oocytes.

### Mitochondrial Ca^2+^ Overload Leads to Mitochondrial Dysfunction in Oocytes

**Figure 2:**
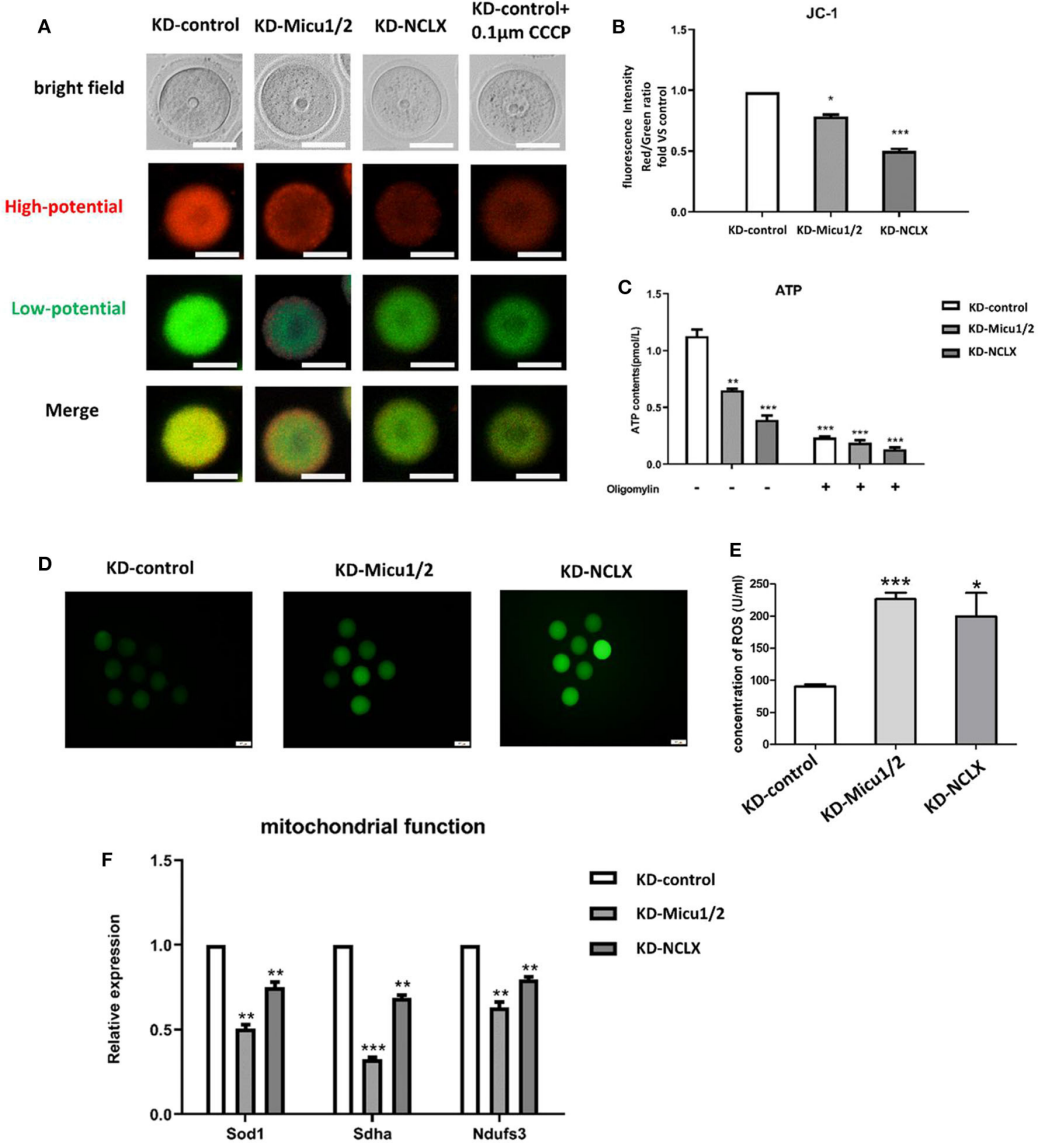
Mitochondrial function is impaired by mitochondrial Ca^2+^ overload. **(A)** Representative images of JC-1 from KD-control, KD-Micu1/2, and KD-NCLX. Scale bar: 50 μm. **(B)** Oocytes from KD-control, KD-Micu1/2, and KD-NCLX were stained with JC-1 and quantification of the relative levels of mitochondrial membrane potential (Δϕm) in oocytes (*n* = 50 for each group). **(C)** ATP (pM) concentrations were evaluated in individual oocytes from KD-control, KD-Micu1/2, and KD-NCLX (*n* = 30 for each group). **(D)** Representative images of CM-H2DCFDA fluorescence (green) in germinal vesicle (GV) stage oocytes from KD-control, KD-Micu1/2, and KD-NCLX. Scale bar: 50 μm. **(E)** Quantification of the relative levels of reactive oxygen species (ROS) in oocytes from KD-control, KD-Micu1/2, and KD-NCLX (U/ml). **(F)** Expression levels of genes involved in mitochondrial function (*Ndufs3, Sdha*, and *Sod1*) in GV-stage oocytes were reduced with mitochondrial Ca^2+^ overload (*n* = 30 for each group). Student's *t-*test and one-way ANOVA were utilized for statistical analyses. *P < 0.05; **P < 0.01; ***P < 0.001. Error bars indicate SEM.

Maturation of the oocyte is a complex progress dependent on mitochondria (Wakai et al., 2012). Previous research has shown that mitochondrial Ca^2+^ homeostasis plays an important role in regulating energy metabolism and several complexes of the electron transport chain (Luongo et al., 2017). Mitochondrial membrane potential (MMP) is an indicator of mitochondrial function, so we used JC-1 to quantify MMP levels in mouse oocytes (Figures 2A,B). Additionally, mitochondria are important organelles that generate ATP in eukaryotes (Deguchi et al., 2015), if the function of mitochondrial is damaged, ATP level may be affected. To determine whether mitochondrial Ca^2+^ levels impair mitochondrial generated ATP, we used an ATP Assay Kit to quantify ATP levels. As expected, both KD-Micu1/2 and KD-NCLX decreased the MMP level and ATP level (Figures 2B,C), which indicated that mitochondrial Ca^2+^ overload leads to mitochondrial dysfunction in oocytes.

Recent studies have shown that mitochondrial reactive oxygen species (ROS) can mediate intracellular signaling (Formentini et al., 2017). Mitochondria are largely responsible for ROS production following egg activation/fertilization (Zorov et al., 2014; Moloney and Cotter, 2018). Since mitochondrial function is associated with oxidative stress, we further evaluated whether mitochondrial Ca^2+^ overload influences ROS production. ROS production in mitochondria is also known to be regulated by MMP (Dai et al., 2018). Intracellular ROS is thought to be a mediator of the cellular signaling in the maintenance of physiological functions (Schieber and Chandel, 2014). To evaluate mitochondrial dysfunction in oocytes we measured intracellular ROS using dihydroethidium and enzyme linked immunosorbent assay. As shown in Figure 2D, quantitative analysis revealed that the relative fluorescence mean intensity in KD-Micu1/2 and KD-NCLX oocytes was significantly higher than control, which was same as the concentration of ROS in the KD-Micu1/2 and KD-NCLX oocytes (Figure 2E). We further detected mitochondrial function related genes such as *Sdha, Nduf3*, and the mitochondrial ROS scavenger *Sod1*. Expression of *Sod1, Sdha*, and the mitochondrial ROS scavenger *Ndufs3* (Figure 2F) were all significantly decreased in mitochondrial Ca^2+^ overloaded oocytes. As expected, CGP-37157 similarly impaired mitochondrial function (Supplementary Figures 4A–E).

### Mitochondrial Ca^2+^ Overload Delays Meiotic Maturation

Germinal vesicle breakdown (GVBD) refers to the dissolution of the nucleus of an oocyte that is arrested in prophase of meiosis I and acts as an indicator of oocyte maturation (Norris et al., 2009). GVBD indicates a resumption of meiosis and the extrusion of the first polar body (PBE) indicates completion of the first meiotic division in oocytes; then, the oocyte will arrest in meiosis II at the metaphase in a pre-fertilization stage (Poueymirou and Schultz, 1987). Therefore, GVBD and PBE usually are used to evaluate the quality of oocytes maturation. Our previous studies have shown that calcium-mediates oocytes maturation disturbance (Deguchi et al., 2015), therefore, we hypothesized that mitochondrial calcium overload may affect meiotic maturation.

**Figure 3:**
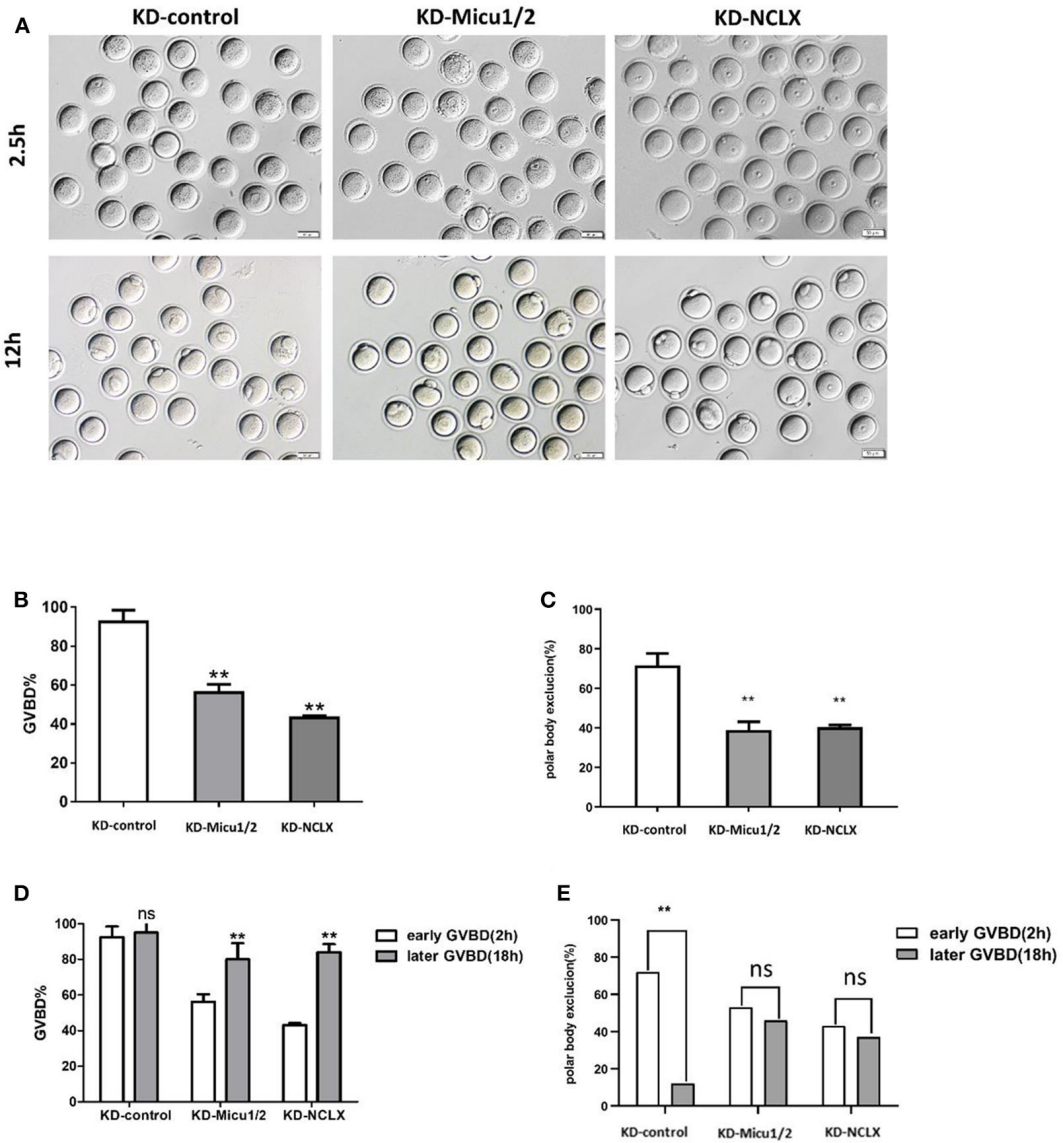
Oocytes maturation is impaired by mitochondrial Ca^2+^ overload. **(A)** Representative images of germinal vesicle breakdown (GVBD) (2.5 h) and the first polar body (PB1) (12 h) extrusion oocytes from KD-control, KD-Micu1/2, and KD-NCLX. Scale bar: 50 μm. **(B)** The percentage of oocytes that successfully progressed to the GVBD during *in vitro* culture in 2.5 h (*n* = 158 for KD-control, *n* = 146 for KD-Micu1/2, and *n* = 132 for KD-NCLX). **(C)** The percentage of oocytes that successfully extracted the first polar body during *in vitro* culture in 12 h (*n* = 115 for KD-control, *n* = 107 for KD-Micu1/2, and *n* = 98 for KD-NCLX). **(D)** Percentage of germinal vesicle breakdown (GVBD) at 2 and 18 h after milrinone removal (Early GVBD *n* = 103 for KD-control, *n* = 43 for KD-Micu1/2, and *n* = 45 for KD-NCLX; Late GVBD *n* = 5 for KD-control, *n* = 67 for KD-Micu1/2, and *n* = 78 for KD-NCLX). **(E)** Polar body extrusion rate of Early or late GVBD: respectively, before or after 2 h after milrinone removal. (Early GVBD *n* = 72 for KD-control, *n* = 53 for KD-Micu1/2, and *n* = 43 for KD-NCLX; Late GVBD *n* = 12 for KD-control, *n* = 46 for KD-Micu1/2, and *n* = 37 for KD-NCLX). Student's *t-*test and one-way ANOVA were utilized for statistical analyses. **P < 0.01. ns, non-significant (*P* > 0.05). Error bars indicate SEM.

As we expected, compared with control oocytes, more KD-Micu1/2 and KD-NCLX oocytes remained at the germinal vesicle stage (Figures 3A,B), and the rate of extrusion of the first polar body was significantly deceased in KD-Micu1/2 or KD-NCLX oocytes 12 h after milrinone removal (Figures 3A,C).

To confirm that mitochondrial Ca^2+^ overload delays or blocks oocyte maturation, we observed oocytes that had completed GVBD within 2 h (early GVBD) or between 2 and 18 h after milrinone removal (late GVBD) and determined the capacity of oocytes to extrude the PB1. In controls, PB1 extrusion efficiency was dramatically deceased for those oocytes that had gone through late GVBD. In addition, we found a notable decease for early vs. late GVBD from KD-Micu1/2 and KD-NCLX oocytes suggesting that those oocytes are delayed in GVBD and PB1 extrusion (Figures 3D,E). Similarly, CGP-37157 also delayed GVBD and PB1 extrusion to impair meiotic maturation (Supplementary Figures 4F–H). We demonstrated that mitochondrial Ca^2+^ overload delayed, but might not arrest meiotic maturation.

### Mitochondrial Ca^2+^ Overload Leads to Impaired PKA Signaling

**Figure 4:**
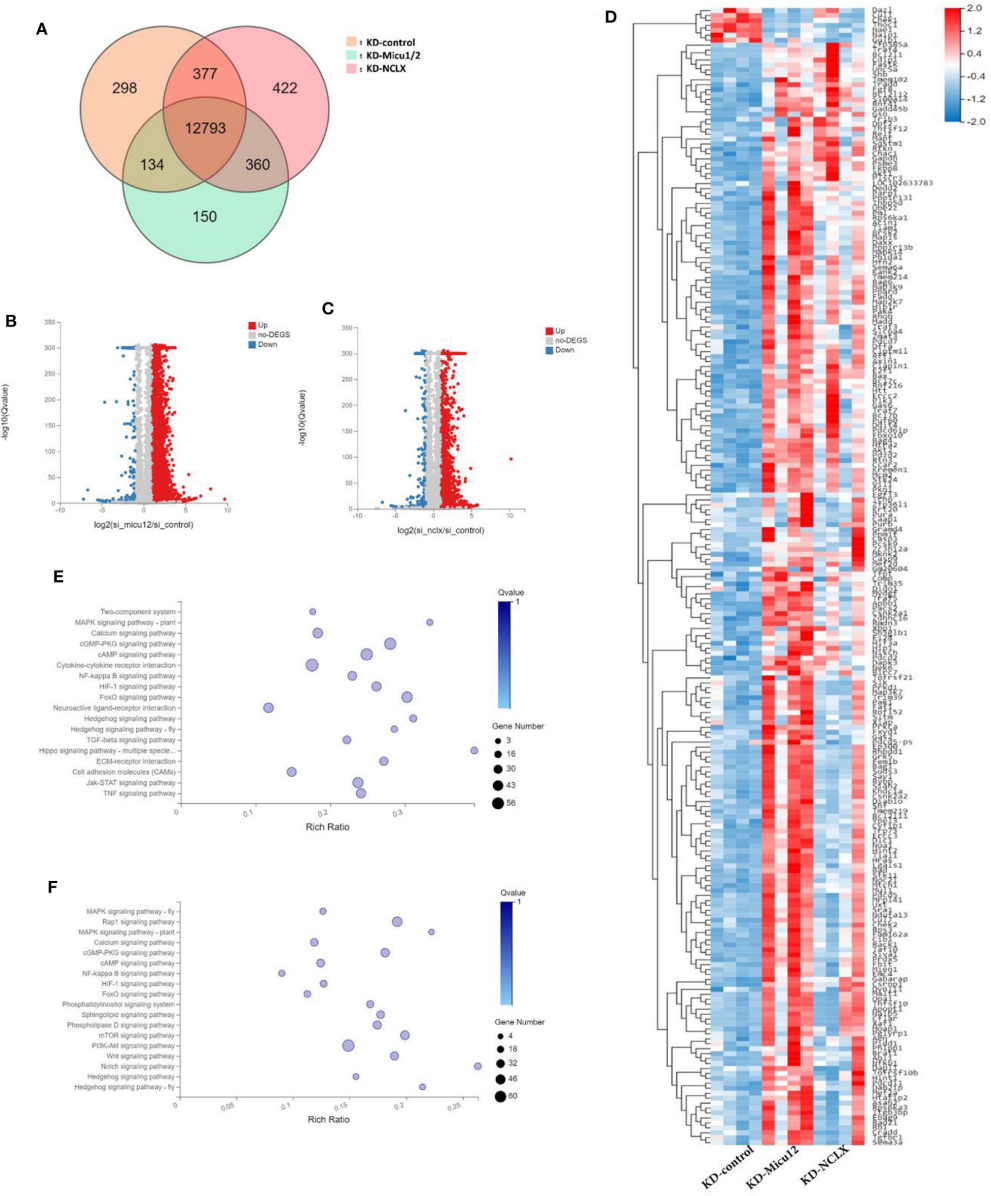
Gene expression changes upon KD-Micu1/2, KD-NCLX. **(A)** VENN showing differential genes in KD-Micu1/2 and KD-NCLX GV oocytes. **(B)** Volcano plot showing upregulated (red) and downregulated (blue) genes in KD-Micu12 GV oocytes. **(C)** Volcano plot showing upregulated (red) and downregulated (blue) genes in KD-NCLX GV oocytes. **(D)** Heatmap showing average expression for all replicates and relative expression between replicates for genes with cell cycle, division functions and mitochondrial functions (based on GO-term). **(E)** Go cellular component showing the biological process and function of the differential genes participated in KD-Micu1/2 GV oocytes (based on KEGG enrichment analysis). **(F)** Go cellular component showing the biological process and function of the differential genes participated in KD-Micu1/2 GV oocytes (based on KEGG enrichment analysis).

**Figure 5:**
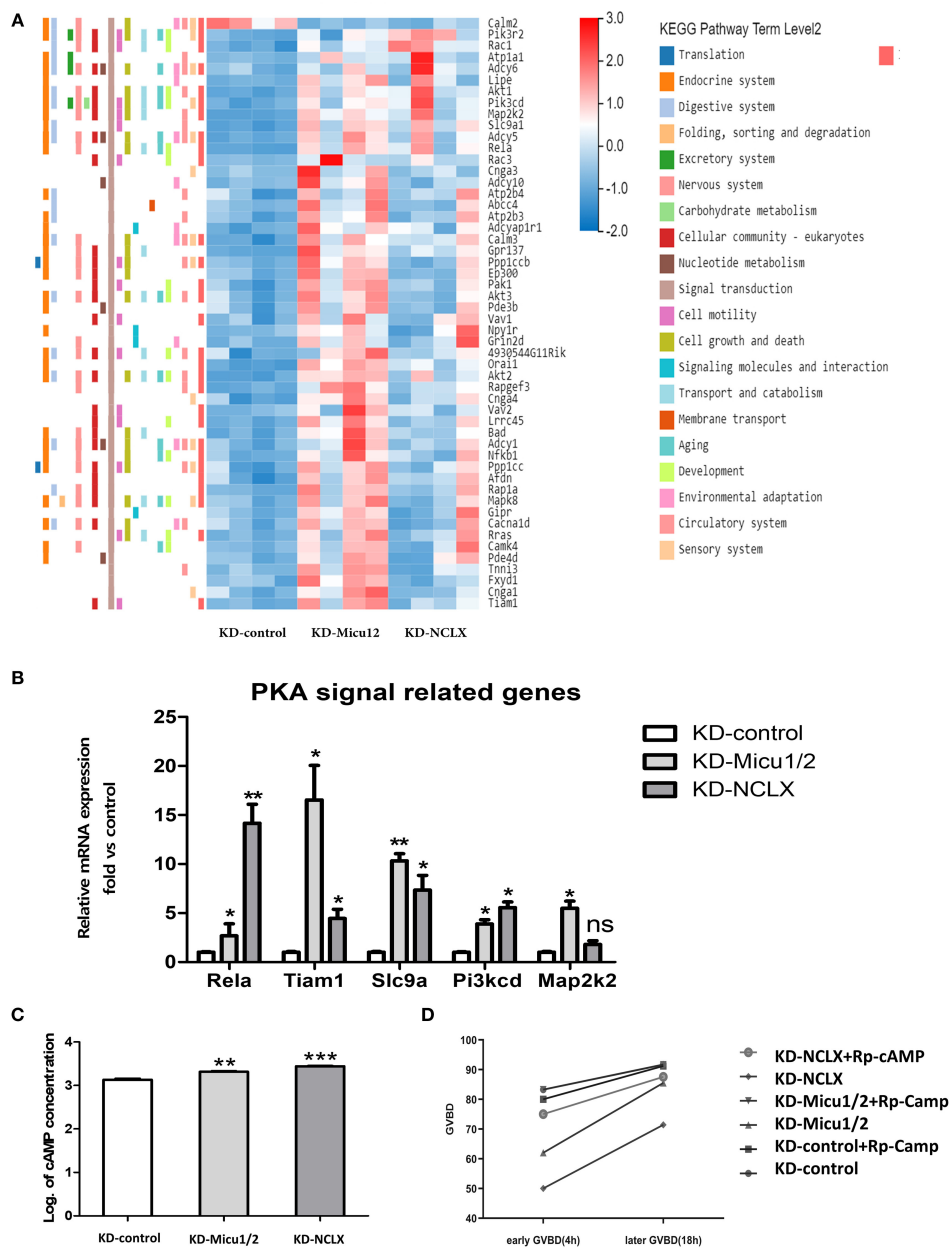
Mitochondrial Ca^2+^ overload leads to meiotic delays which may related to impairing PKA signaling. **(A)** Heatmap showing the average expression for all replicates and relative expression between replicates for genes with PKA signal pathway (based on GO term). **(B)** Dysregulation of genes associated with PKA pathway signaling in KD-control, KD-Micu1/2, and KD-NCLX oocytes (*n* = 30 for each group). **(C)** Quantification of the relative levels of cAMP in oocytes. (*n* = 40 for each group). **(D)** Percentage of GVBD at 2.5 h and 18 h after milrinone removal for oocytes treated with or without Rp-cAMP. (*n* = 24 for KD-control, *n* = 30 for KD-control+ Rp-cAMP, *n* = 24 for KD-Micu1/2, *n* = 24 for KD-Micu1/2+ Rp-cAMP, and *n* = 40 for KD-NCLX, *n* = 32 for KD-NCLX+ Rp-cAMP). Student's *t-*test and one-way ANOVA were utilized for statistical analyses. *P < 0.05, **P < 0.01, ***P < 0. 001; ns, non-significant (*P* > 0.05). Error bars indicate SEM.

Given the known mitochondrial Ca^2+^ overload leads to mitochondrial dysfunction and meiotic maturation, the mechanism of delayed meiosis is still unknown. We anticipated that KD-Micu1/2 and KD-NCLX during oocyte growth would alter expression of genes controlling meiotic maturation. Then we used RNA-seq to discover underlying mechanisms caused by mitochondrial calcium overload. KD-control, KD-Micu1/2 and KD- NCLX data sets have 13,602, 13,952, and 13,437 genes totally (Figure 4A). As shown in volcano plot (Figures 4B,C), there are 5,272 genes upregulated (red) and 370 genes downregulated (blue) genes in KD-Micu1/2 GV oocytes, and 2,774 genes upregulated (red) and 348 genes downregulated (blue) genes in KD-NCLX GV oocytes. And we used heatmap to show average expression for all replicates and relative expression between replicates for genes with cell cycle, division functions and mitochondrial functions (Figure 4D). KEGG Pathway Analysis indicated that some pathway participated in metabolism and cAMP-related signal cascades are highly variable (Figures 4E,F). Interestingly, using KEGG Pathway Analysis, we found some genes belonging to the PKA signaling pathway were dysregulated in KD-Micu1/2 and KD-NCLX oocytes (Figure 5A). It is well-known that PKA maintains meiotic arrest in response to high levels of cAMP, such that meiotic arrest in prophase is normally maintained by high levels of cAMP that activates PKA signal that in turn down regulates the mature promoting factor (MPF) activity (Poueymirou and Schultz, 1987; Norris et al., 2009). Then we use quantitative Real-Time PCR to verified the results (Figure 5B). As shown in Figure 5C, levels of cAMP in KD-Micu1/2 and KD-NCLX oocytes dramatically increased. To further confirm that PKA/cAMP signaling is involved in the GVBD delay observed in mitochondrial Ca^2+^ overloaded oocytes, we inhibited PKA/cAMP signaling with cAMP antagonist 8-bromo-Rp-cAMP (Rp-cAMP) (Figure 5D). After removal of milrinone, Rp-cAMP treatment did not affect the high rate of GVBD in control oocytes. Meanwhile, in KD-Micu1/2 and KD-NCLX oocytes, we observed a significant alleviation the GVBD delay. These results suggest that the GVBD delay observed in mitochondrial Ca^2+^ overloaded oocytes which has showed in Figure 3D may be due to the abnormal activation of the PKA/cAMP signaling pathway.

### Ru360 Can Ameliorate Mitochondrial Dysfunction and Recover Oocyte Maturation Caused by Mitochondrial Ca^2+^ Overload

Mitochondrial dysfunction and meiosis maturation defects had been observed in KD-Micu1/2 and KD-NCLX oocytes, but whether impaired oocyte mitochondrial function can be improved by decreasing mitochondrial Ca^2+^ level is unclear. As Ru360 is a well-known inhibitor of the mitochondrial Ca^2+^ uniporter MCU (Paillard et al., 2018), we next examined whether Ru360 treatment could improve mitochondrial function in oocytes. To this end, we supplemented the *in vitro* maturation (IVM) solution for KD-Micu1/2 and KD-NCLX oocytes with 0, 5, 10, and 20 μM Ru360 and analyzed mitochondrial Ca^2+^ levels by Rhod-2 AM staining. Quantitative analysis of relative fluorescence mean intensity reveals that KD-Micu1/2 and KD-NCLX oocytes increased mitochondrial Ca^2+^ levels, and the addiction of 5 μM Ru360 downregulated these mitochondrial Ca^2+^ levels to those seen in control oocytes (Supplementary Figures 5A,B).

**Figure 6:**
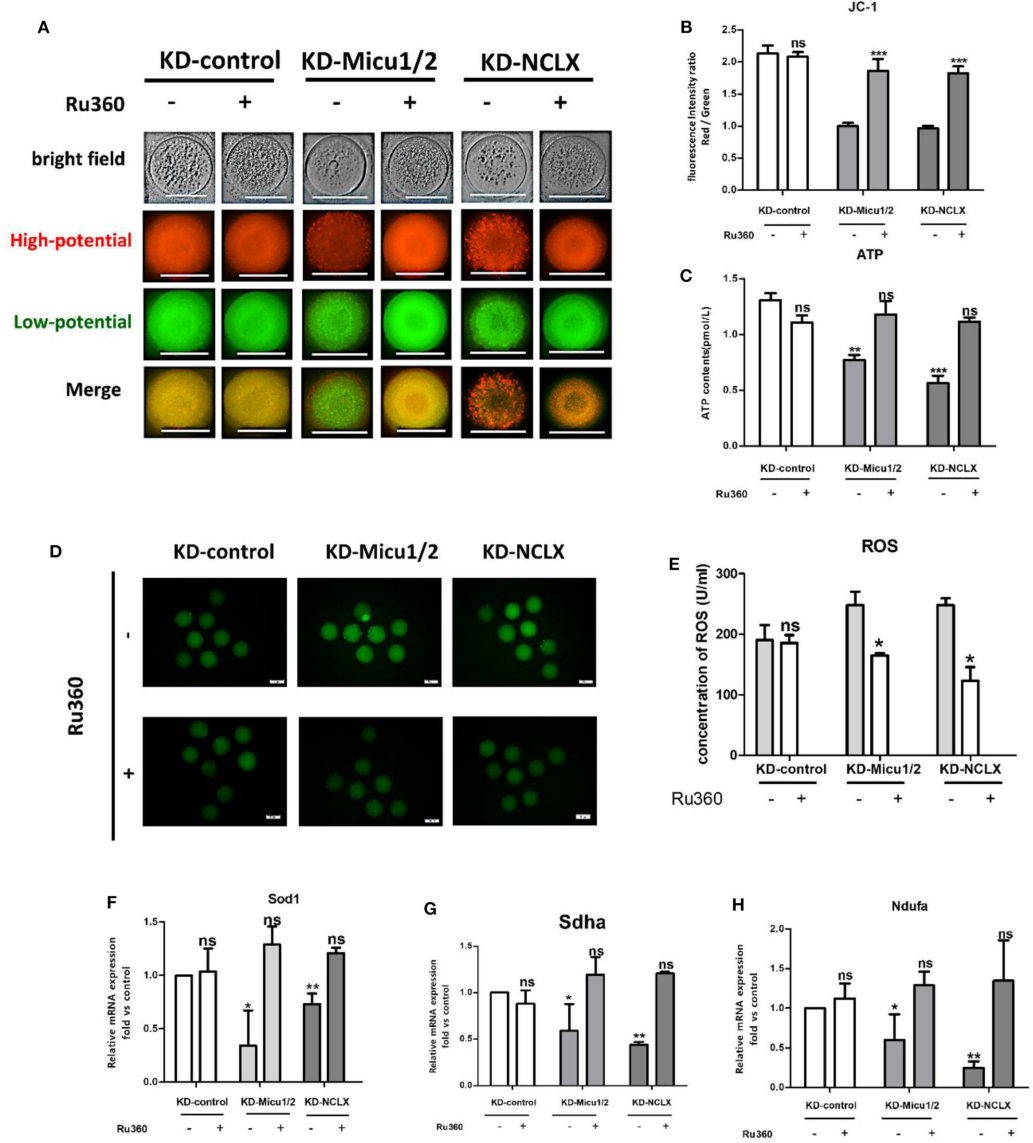
Mitochondrial dysfunction and recovery of oocyte maturation by decreasing mitochondrial Ca^2+^ levels. **(A)** Image of oocytes from KD-control, KD-control+Ru360, KD-Micu1/2, KD-Micu1/2+Ru360, KD-NCLX and KD-NCLX+Ru360 were stained with JC-1. **(B)** Oocytes from KD-control, KD-control+Ru360, KD-Micu1/2, KD-Micu1/2+Ru360, KD-NCLX, and KD-NCLX+Ru360 were stained with JC-1 and quantification of the Red/Green ratio of mitochondrial membrane potential (Δϕm) in oocytes (*n* = 30 for each group). **(C)** ATP (pM) concentrations were evaluated in individual oocytes from KD-control, KD-control+Ru360, KD-Micu1/2, KD-Micu1/2+Ru360, KD-NCLX, and KD-NCLX+Ru360 (*n* = 30 for each group). **(D)** Representative images of CM-H2DCFDA fluorescence (green) in germinal vesicle (GV) stage oocytes from KD-control, KD-control+Ru360, KD-Micu1/2, KD-Micu1/2+Ru360, KD-NCLX, and KD-NCLX+Ru360. Scale bar: 50 μm. **(E)** Quantification of the relative levels of ROS in oocytes. (*n* = 35 for each group). **(F–H)** Expression levels of genes involved in mitochondrial function (*Ndufs3, Sdha*, and *Sod1*) in GV-stage oocytes from KD-control, KD-control+Ru360, KD-Micu1/2, KD-Micu1/2+Ru360, KD-NCLX, and KD-NCLX+Ru360 (*n* = 30 for each group). Student's *t-*test and one-way ANOVA were utilized for statistical analyses. *P < 0.05, **P < 0.01, ***P < 0. 001; ns, non-significant (*P* > 0.05). Error bars indicate SEM.

We next examined mitochondrial Ca^2+^ overload in oocytes with 5 μM Ru360 and analyzed the ROS, ATP level, and Δϕm levels as well as the mRNA expression levels of mitochondrial genes *Ndufs3, Sdha*, and *Sod1*. As shown in Figures 6A,B, Ru360 reversed the decreased MMP levels in KD-Micu1/2 or KD-NCLX oocytes. Mitochondria depend on calcium signals to maintain their function, especially for their capacity to synthesize ATP. As we expected, the ATP level decreased with increasing mitochondrial Ca^2+^ levels (Figure 6C). High levels of ROS caused by the mitochondrial respiratory chain in oocytes was mediated by mitochondrial Ca^2+^ levels (Figures 6D,E). Moreover, the mRNA expression of mitochondrial function genes *Ndufs3, Sdha*, and *Sod1* also distinctly increased after treatment with Ru360 (Figures 6F–H). As mitochondrial function is closely associated with meiosis competence in oocytes, we further evaluated the quality of oocytes. As shown in Table 1, the rates of GVBD and PB1 significantly increased after mitochondrial Ca^2+^ deceased, which suggested recovery of meiosis competence. Collectively, the results suggest that *in vitro* administration of Ru360 could improve mitochondrial dysfunction and meiosis defect in mitochondrial Ca^2+^ overload oocytes.

**Table 1:** Effect of Ru360 treatment on recover meiosis maturation of mitochondrial calcium overload oocytes.

**Groups**	**No. of oocytes culture**	**No. of oocytes GVBD (%, mean ± SEM)**	**No. of oocytes PB1 (%, mean ± SEM)**
KD-control	125	116 (92.67 ± 2.64092)^c^	87 (84.6000 ± 3.76076)^c^
KD-control+Ru360	128	117 (91.0600 ± 1.22676)^c^	100 (86.0667 ± 3.28346)^c^
KD-Micu1/2	155	114 (73.7333 ± 3.64478)^a^	96 (59.0667 ± 2.41822)^b^
KD-Micu1/2+Ru360	140	128 (91.95 ± 0.98489)^c^	101 (80.4333 ± 4.95356)^c^
KD-NCLX	116	80 (69.1333 ± 0.46667)^a^	40 (44.2000 ± 5.29371)^a^
KD-NCLX+Ru360	123	112 (91.7167 ± 1.23029)^c^	98 (83.9000 ± 3.25628)^c^

a−c *Significant difference in the same column (P < 0.05); SEM, standard error of the mean*.

### Declining Mitochondrial Ca^2+^ Rescues Mitochondrial Dysfunction and Meiosis Maturation in Oocytes of Obese Mice

**Figure 7:**
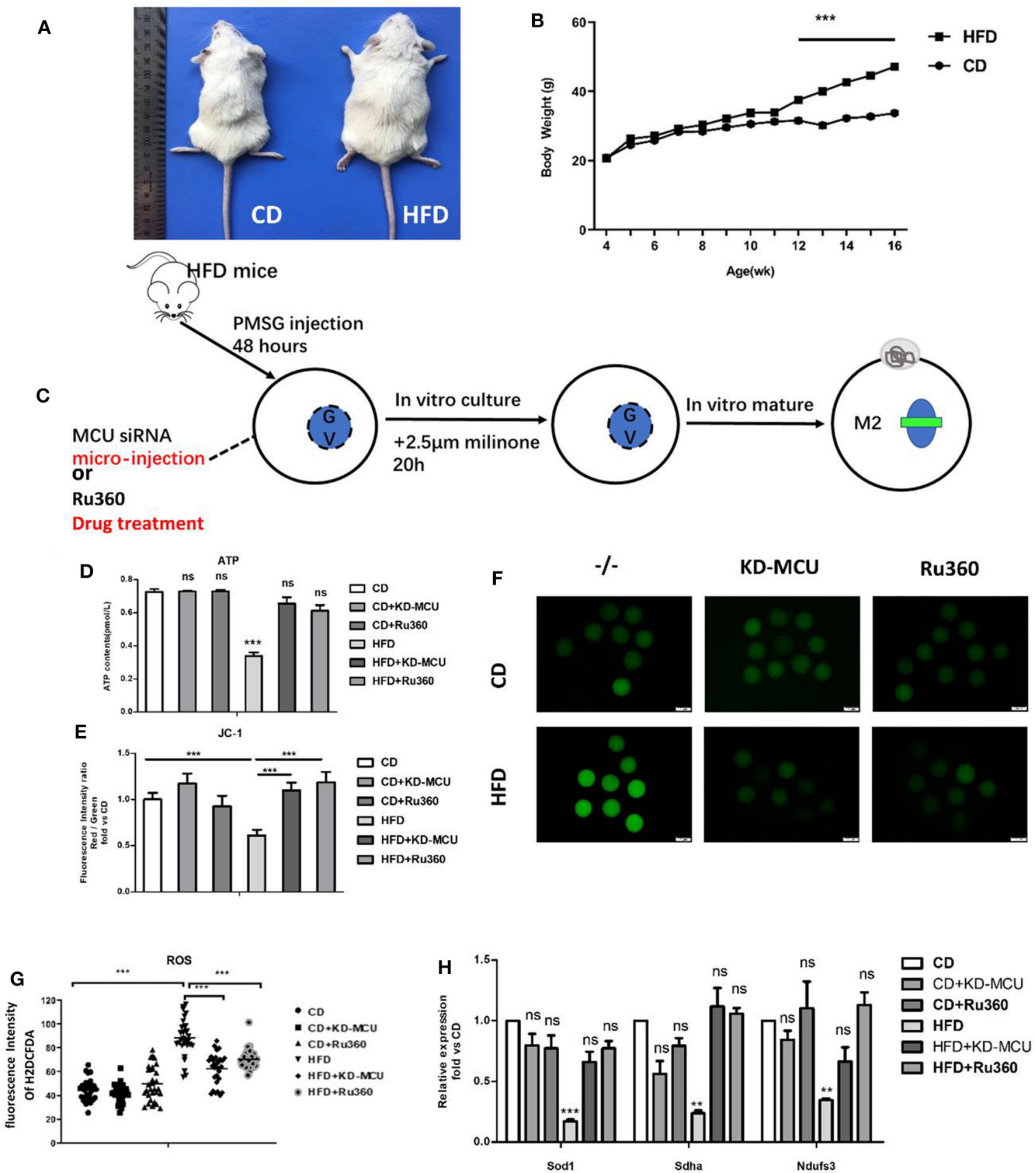
Declining mitochondrial Ca^2+^ rescues mitochondrial dysfunction in obese oocytes. **(A,B)** Female mice receiving a control diet (CD) or high-fat diet (HFD) for 13 weeks were evaluated for body weight (*n* = 22 for each group). **(C)** The process about declining mitochondrial Ca^2+^ in obese mouse oocytes by microinjection special siRNA sequence to knockdown expression about MCU or special inhibitor of MCU. **(D)** ATP (pM) concentrations were evaluated in individual oocytes from CD, CD+KD-MCU, and CD+Ru360 mice and HFD, HFD+KD-MCU, and HFD+Ru360 mice (*n* = 30 for each group). **(E)** Oocytes from CD, CD+KD-MCU, and CD+Ru360 mice and HFD, HFD+KD-MCU, and HFD+Ru360 were stained with JC-1 and quantification of the relative levels of mitochondrial membrane potential (Δϕm) in oocytes (*n* = 30 for each group). **(F)** Representative images of CM-H2DCFDA fluorescence (green) in germinal vesicle (GV) stage oocytes from CD, CD+KD-MCU, CD+Ru360 mice and HFD, HFD+KD-MCU, and HFD+Ru360 mice. Scale bar: 50 μm. **(G)** Quantification of the relative levels of ROS in oocytes from CD, CD+KD-MCU, and CD+Ru360 mice and HFD, HFD+KD-MCU, and HFD+Ru360 (*n* = 35 for each group). **(H)** Expression levels of genes involved in mitochondrial function (*Ndufs3, Sdha*, and *Sod1*) in HFD oocytes were rescued by declining mitochondrial Ca^2+^ level (*n* = 30 for each group). Student's *t-*test and one-way ANOVA were utilized for statistical analyses. **P < 0.01; ***P < 0.001; ns. means non-significant (*P* > 0.05). Error bars indicate SEM.

We have shown a meiosis maturation defect in GVBD from mitochondrial Ca^2+^ overload oocytes, which may be a consequence of mitochondrial dysfunction. Further, the capacity of impaired mitochondrial could be recovered by decreasing mitochondrial Ca^2+^ levels. Therefore, we examined whether the beneficial effects of decreasing mitochondrial Ca^2+^ levels could also ameliorate deficient mitochondrial function in oocytes of high fat diet (HFD) mice. Firstly, we established an obesity model by feeding HFD diet 12 weeks constantly (Figures 7A,B). And then GV-stage oocytes from control diet (CD) mice, HFD mice, and HFD mice infected with an siRNA targeting MCU (HFD + KD-MCU), and HFD mice administered Ru360 (HFD+Ru360) (Figure 7C). All of these groups were analyzed for ROS, ATP, and Δϕm levels as well as the mRNA expression level of mitochondrial function genes *Ndufs3, Sdha*, and *Sod1*. As shown in Figures 7D–H, the data suggested that downregulating mitochondrial Ca^2+^ levels promoted the potential of mitochondrial to improve quality of oocytes derived from obese mice. As mitochondrial function was closely associated with ATP content and oocyte quality, we further examined whether mitochondrial Ca^2+^ levels influence meiosis maturation. GV-stage oocytes from CD, HFD, HFD+ KD-MCU, and HFD+Ru360 mice were analyzed for frequency of GVBD and PB1 extrusion. The rates of GVBD and PB1 extrusion significantly increased after mitochondrial Ca^2+^ decrease, which suggested recovery of obese oocyte quality (Figures 7A–D). These observations indicated that proper downregulation of mitochondrial Ca^2+^ concentration could attenuate meiotic defects in damaged oocytes from obese mice.

## Discussion

Obesity or diabetes—regarded as global health problems (Ou et al., 2019)—are common among women of reproductive age (Atzmon et al., 2017). Obese women take longer to conceive, and, even in assisted reproduction, they have lower implantation, pregnancy, and miscarriage rates. Therefore, many studies have focused on oocyte quality from obese women. Mitochondria are energy factories for many cellular processes, such as synthesizing ATP and heat production, which are essential for meiotic maturation and postembryonic development (Babayev and Seli, 2015). For this reason, research has attempted to improve deficient mitochondria from oocytes to maintain the potential for reproduction. Studies have indicated that transplanting mitochondria from healthy oocytes to severely obese patients dramatically increased their implantation and pregnancy rates (Machtinger et al., 2012; Babayev and Seli, 2015). Recent evidence suggests that supplementation with co-enzyme Q10 (CoQ10), melatonin, or glutathione (GSH) can improve oocyte mitochondrial abnormalities (Ben-Meir et al., 2015). Taken together, these results show that mitochondrial dysfunction can be caused by obesity and indicates that mitochondrial condition is critical for oocyte maturation and reproduction. However, the mechanisms accounting for this phenomenon are still not clear. To determine a possible mechanism for how obesity affects oocyte quality, we focused on the mechanisms regulating mitochondrial function.

In the study of somatic cells, mitochondrial Ca^2+^ overload contributes to impaired cardiac function thereby causing myocardial infarction (Santulli et al., 2015). Mitochondrial Ca^2+^ homeostasis has become an important biological problem as it is crucial to several pathologies as well as regulation of cytoplasmic redox state, signal transduction, regulation of chromosomal defects, and maturation and fertilization in human oocytes (Krishnamoorthy et al., 2006). Therefore, it is possible that overload in mitochondrial Ca^2+^ regulation could have a negative effect on meiosis maturation and oocyte development. Our study clearly demonstrates that mitochondrial Ca^2+^ overload leads to abnormalities in mitochondrial function and meiosis maturation. Here, we confirmed the importance of mitochondrial Ca^2+^ levels by two knockdown methods targeting Micu1/2 and NCLX by infecting siRNAs targeting these mRNA into mouse oocytes. We found that mitochondrial Ca^2+^ overload could give rise to a series of problems such as delayed meiosis maturation, depleted oocyte mitochondrial gene expression, and impaired mitochondrial function and all these changes could be reversed by decreasing the level of mitochondrial Ca^2+^. These results show that decreasing mitochondrial Ca^2+^ levels can improve mitochondrial function, meiosis maturation, and expression of PKA signaling-related genes (Nishimura et al., 2014), which is similar in our previous study that in bovine oocytes (Hu et al., 2018).

Previous studies have shown that there are two different situations that can affect meiosis maturation (Eymery et al., 2016). To confirm that mitochondrial Ca^2+^ overload can delay or block oocyte maturation, we analyzed the rate of GVBD and PB1 in control and mitochondrial Ca^2+^ overloaded oocytes at different time points after the removal of milrinone.

Given the important role of mitochondrial Ca^2+^ in regulating mitochondrial function, we evaluated the effect of mitochondrial function from different aspects. We show that overload of mitochondrial Ca^2+^ is accompanied by mitochondrial dysfunction associated with increased oxidative phosphorylation and reduced ATP levels. Since that the bioenergetic state of an oocyte depends on MMP (Wilding et al., 2001), we stained oocytes with JC-1 to quantify the relative levels of Δϕm. As we expected, mitochondrial Ca^2+^ overload caused a significant reduction of Δϕm in oocytes, which indicates that the action of the electron transfer chain (ETC)—a critical mitochondrial function—was impaired. Genes *Ndufs3, Sdha*, and *Sod1* have been reported to play a role in mitochondrial function (Gibson et al., 2005). Gene expression analysis with qRT–PCR of GV-stage oocytes revealed a significant decrease in *Ndufs3, Sdha*, and *Sod1* expression, which suggested that mitochondrial function was damaged by mitochondrial Ca^2+^ overload.

We have shown that mitochondrial Ca^2+^ plays a key role in the adaptive mechanisms that allow mitochondria to perform essential functions. This finding raises the possibility that decreasing mitochondrial Ca^2+^ to proper levels in obese oocytes can recover defects in mitochondrial function to, ultimately, rescue meiosis maturation. Taken together, these results indicate that proper mitochondrial Ca^2+^ regulation is critical for maintaining mitochondrial function and oocyte maturation. Targeted knockdown or pharmacological inhibition of MCU in obese mouse oocytes led to mitochondrial Ca^2+^ level decease, improvement in mitochondrial function, and recovery of the meiosis maturation. In this study, we demonstrate that proper regulation of mitochondrial Ca^2+^ in an obesity model restored oocyte mitochondrial gene expression and improved mitochondrial activity.

In summary, our study has proved that obese could cause impaired Micu1/2 expression which related to mitochondrial Ca^2+^ homeostasis. Moreover, mitochondrial Ca^2+^ overload in mice oocytes leads to meiosis maturation delay and mitochondrial dysfunction. Our results also highlight the critical role of mitochondrial Ca^2+^ regulation in maintaining mitochondrial function and oocyte maturation. These results enable researchers to find reliable approaches to solve mitochondrial dysfunction of oocytes from obese, aged, or other unideal source, and provide a new molecular pathway to control oocyte development and improve women reproduction.

## Data Availability Statement

The datasets generated for this study can be found in NCBI BioProject ID PRJNA661665.

## Ethics Statement

The animal study was reviewed and approved by The Institutional Animal Care and Use Committee of China Agricultural University.

## Author Contributions

LZ participated in the research design, animal research, data analysis, and writing of the paper. ZW participated in animal research and writing of the paper. TL participated in animal research, siRNA microinjection, and revising of the paper. LM, YL, and XF contributed equally to the paper. LM participated in siRNA microinjection and staining performance. YL participated in data analysis. XF participated in the writing and revising of the paper, provided substantial advice in designing the study and assisting in the division of labor, writing, and revising the paper. All authors contributed to the article and approved the submitted version.

## Conflict of Interest

The authors declare that the research was conducted in the absence of any commercial or financial relationships that could be construed as a potential conflict of interest.
