# Supplementary material for: Mitochondrial Ca2+ Overload Leads to Mitochondrial Oxidative Stress and Delayed Meiotic Resumption in Mouse Oocytes
Source: Front Cell Dev Biol. 2020 Dec 15;8:580876. doi: 10.3389/fcell.2020.580876 (PMC7770107; doi:10.3389/fcell.2020.580876)
Supplement: Supplementary file 1 [file Presentation_1.pdf]

Supplemental information

Supplementary Figure S1

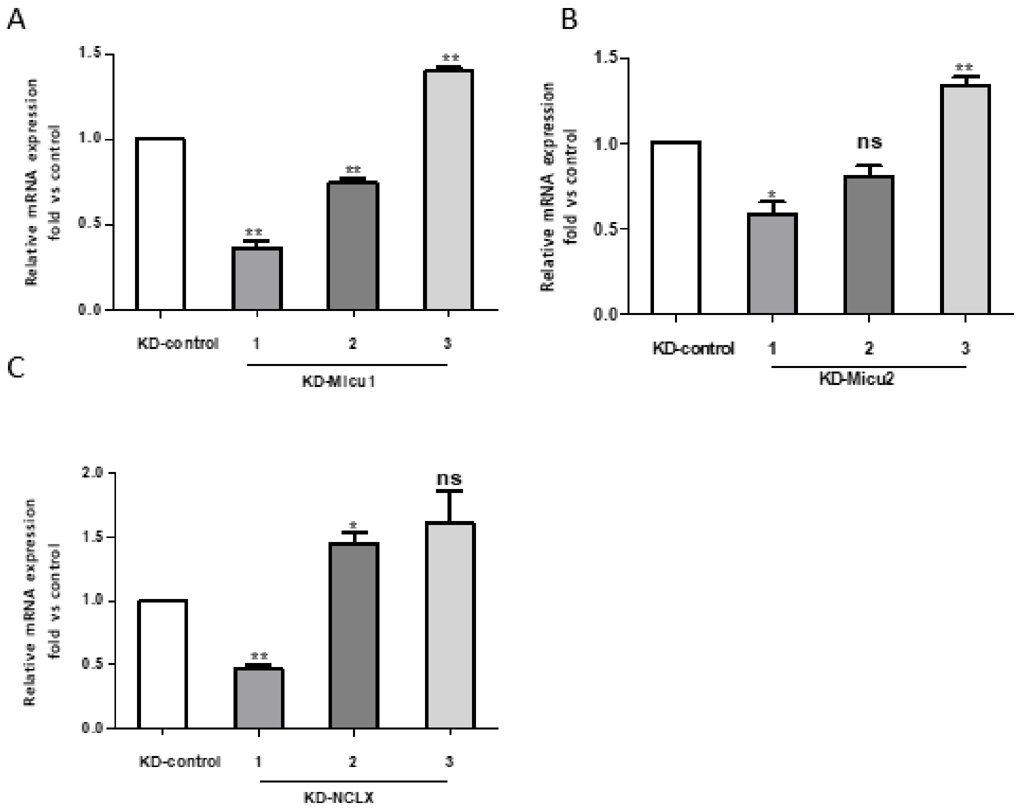

Supplementary Figure S1. Quantitative real-time PCR of efficiency of KD-Micu1, KD-Micu2 and KD-NCLX

Quantitative Real-Time PCR showing (A) Micu1, (B) Micu2, and (C) NCLX expression in control and three interference sequences of Micu1, Micu2, and NCLX oocytes, respectively. (n = 30 for each group)

Student's *t*-test was utilized for statistical analyses. Error bars indicate mean ± SEM. * $P < 0.05$, ** $P < 0.01$ versus control group.

Supplementary Figure S2.

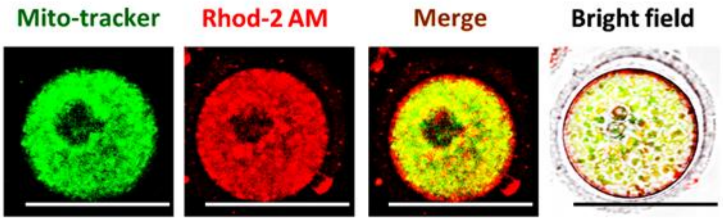

Supplementary Figure S2.

Representative images of co-strained about Rhod-2 AM fluorescence (red)and Mito-tracker(green) in GV oocytes. Scale bar: 50 µm.

Supplementary Figure S3

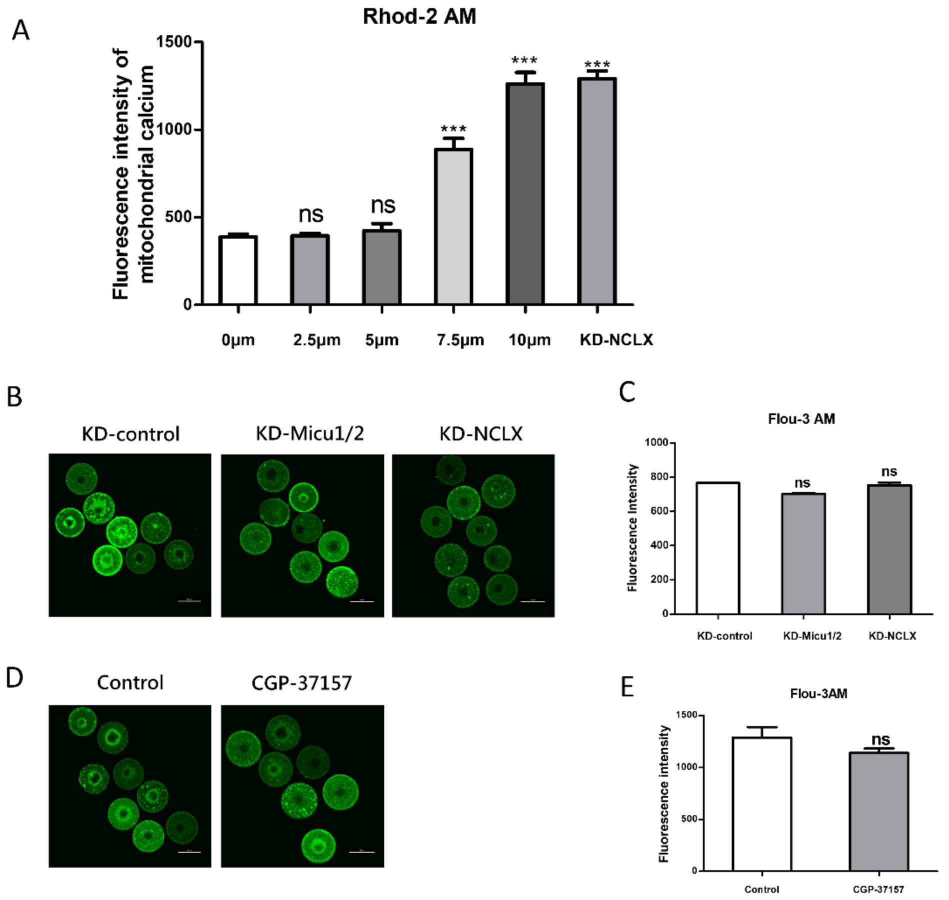

Supplementary Figure S3. Mitochondrial Ca^{2+} level in oocytes from CGP-37157 treated oocytes and cytosolic Ca^{2+} level in oocytes from KD-control, KD-Micu1/2, and KD-NCLX CGP-37157 treatment

(A) Quantification of the relative levels of mitochondrial Ca^{2+} ion in germinal vesicle (GV) stage oocytes from control and different concentration treatment oocytes with CGP-37157 (n = 30 for each group). (B) Representative images of Flou-3 AM fluorescence (green) in GV oocytes from KD-control, KD-Micu1/2, and KD-NCLX. Scale bar: 50 μm. (C) Quantification of the relative levels of cytosolic Ca^{2+} ion in GV oocytes from KD-control, KD-Micu1/2, and KD-NCLX mouse. (n =30 for each group). (D) Representative images of Flou-3 AM fluorescence

(red) in GV oocytes from control and CGP-37157 treatment mouse. Scale bar: 50 µm. (E) Quantification of the relative levels of cytosolic Ca^{2+} ion in GV oocytes from control and CGP-37157 treatment mouse. (n = 30 for each group)

Student's *t*-test was utilized for statistical analyses. Error bars indicate mean ± SEM. ns represent no significant $p>0.05$, *** $P < 0.001$ versus control group.

Supplementary Figure S4

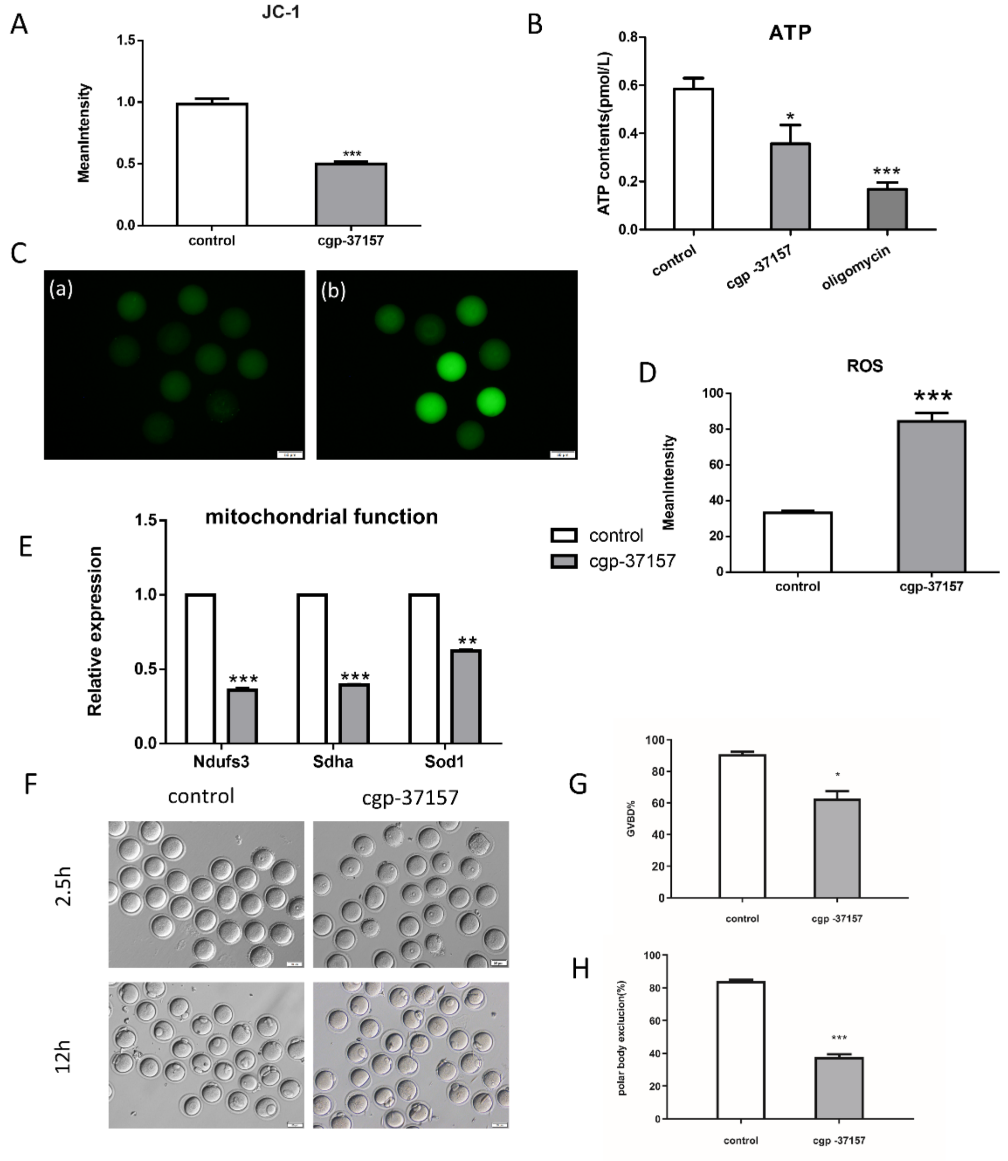

Supplementary Figure S4. Mitochondrial function and meiotic competence of oocytes treated with cgp-37157

(A) Oocytes from control and CGP-37157 treatment were stained with JC-1 and quantification of the relative levels of lyses (n = 50 for each group). (B) ATP (pM) concentrations were evaluated in individual oocytes from control and CGP-37157 treatment (n = 30 for each group).

(C) Representative images of CM-H2DCFDA fluorescence (green) in germinal vesicle (GV) stage oocytes from control and CGP-37157 treatment. Scale bar: 50 μm. (D) Representative images of CM-H2DCFDA fluorescence (green) in oocytes (n = 35 for each group). (E) Expression levels of genes involved in mitochondrial function (*Ndufs3*, *Sdha*, and *Sod1*) in GV oocytes were reduced with CGP-37157 treatment (n = 30 for each group). (F) Representative images of germinal vesicle breakdown (GVBD) (2.5 h) and the first polar body (1PB) (12 h) extrusion oocytes from control and CGP-37157 treatment. Scale bar: 50 μm. (G) The percentage of oocytes that successfully progressed to the GV bread down during *in vitro* culture in 2.5 h. (H) The percentage of oocytes that successfully extracted the first polar body during *in vitro* culture in 12 h.

Student's *t*-test was utilized for statistical analyses. Error bars indicate mean ± SEM. * $P < 0.05$, ** $P < 0.01$, *** $P < 0.001$ versus control group.

Supplementary Figure S5

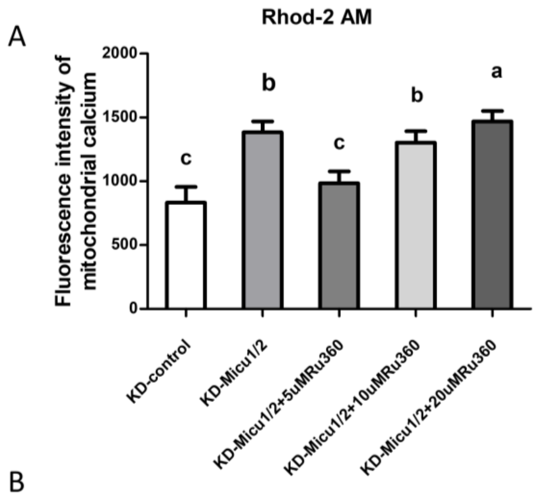

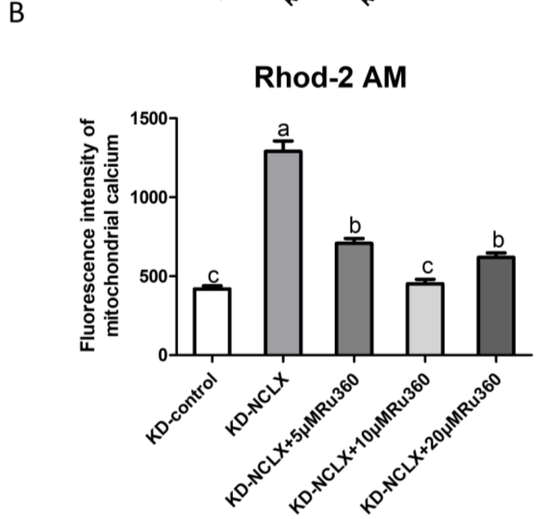

Supplementary Figure S5 (A-B) Quantification of the relative levels of mitochondrial Ca2+ in oocytes different concentration treatment with Ru360 (n = 30 for each group)

Student's one-way ANOVA was utilized for statistical analyses.

Different letters above columns indicate significant differences between groups (*P* <0.05); Error bars indicate mean ± SEM.

Supplementary Figure S6

A

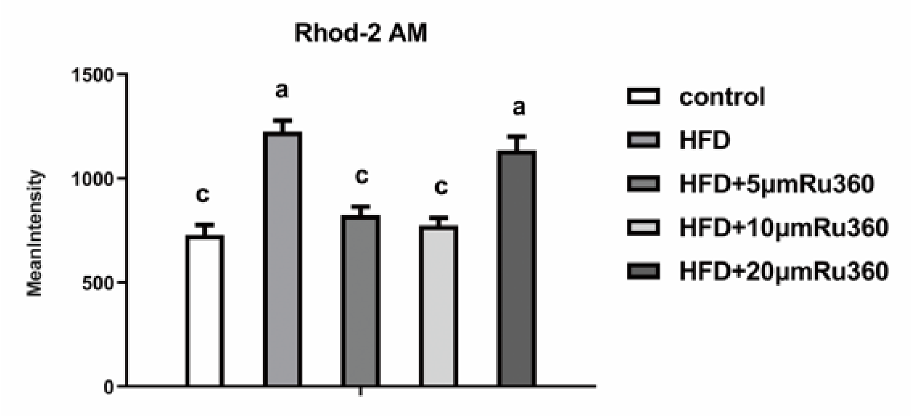

B

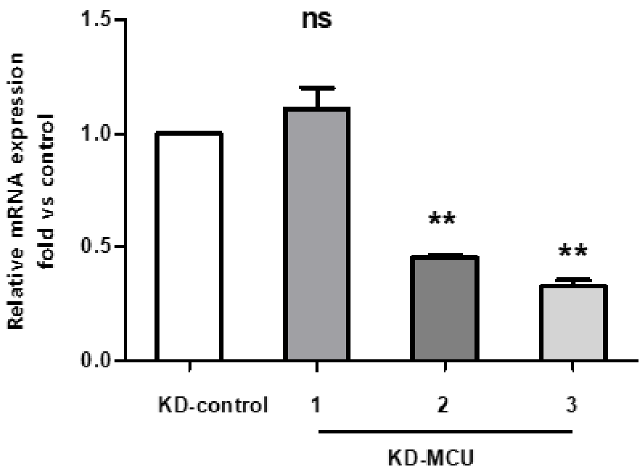

Supplementary Figure S6 (A) Quantification of the relative levels of mitochondrial Ca2+ in HFD oocytes different concentration treatment with Ru360 (n = 30 for each group) (B) Quantitative real-time PCR of efficiency of siRNA MCU Quantitative Real-Time PCR showing expression in control and three interference sequences of MCU oocytes, respectively. (n = 30 for each group)

Student's t-test and one-way ANOVA were utilized for statistical analyses. Different letters above columns indicate significant differences between groups (*P*< 0.05)

***P* < 0.01; Error bars indicate mean ± SEM.

Supplementary Figure S7

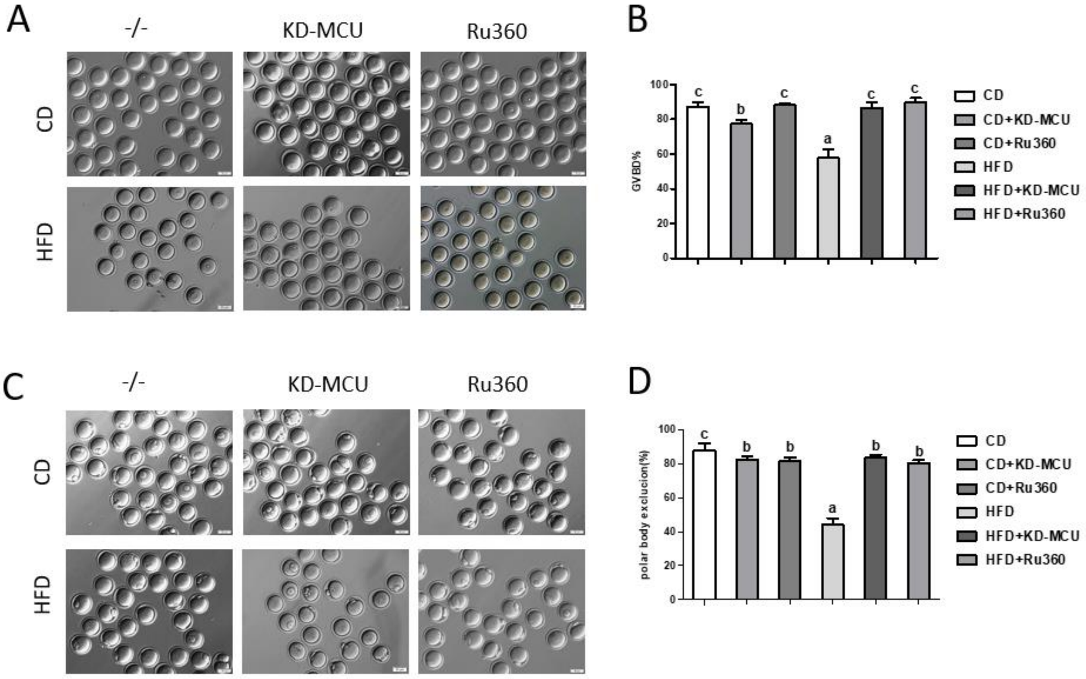

Supplementary Figure S7(A) Representative images of germinal vesicle breakdown (GVBD) (2.5 h) oocytes from CD, CD+KD-MCU, CD+Ru360 and HFD, HFD+KD-MCU, HFD+Ru360. Scale bar: 50 µm. (B) The percentage of oocytes that successfully progressed to GVBD during in vitro culture in 2.5 h. (n = 99 for CD, n = 91 for CD+KD-MCU, n = 91 for CD+Ru360, n = 84 for HFD, n = 104 for HFD+KD-MCU, n = 106 for HFD+Ru360) (C) Representative images of the first polar body (PB1) (12 h) extrusion oocytes from CD, CD+KD-MCU, and CD+Ru360 mice and HFD, HFD+KD-MCU, and HFD+Ru360 mice. Scale bar: 50 µm. (D) The percentage of oocytes that successfully extracted the PB1 during in vitro culture in 12 h. (n = 88 for CD, n = 76 for CD+KD-MCU, n = 76 for CD+Ru360, n = 86 for HFD, n = 78 for HFD+KD-MCU, n = 80 for HFD+Ru360). Student's one-way ANOVA was utilized for statistical analyses. Different superscript letters (a-c) represent a significant difference in the same column. Different letters above columns indicate significant differences between groups ($P < 0.05$). Error bars indicate mean ± SEM.

Supplementary Table 1 Oligonucleotide primer sequences used for quantitative Real-Time PCR

Gene	Primer sequences (5’-3’)	Product size(bp)	GenBank accession number or Reference
Micu1	F: ACACCCTCAAGTCTGGCTTAT	268	NM-144822
	R: TTCCCATCTTGAAGTGCTTCTT		
Micu2	F: TCGGCGCAGAAAAATTATTTGG	106	NM-028643
	R: GTGTCATGTAATACTCTCCGTCG		
NCLX	F: CTGGAGCCAGACGGATTACTG	225	NM-133221
	R: CCACACCTCTAGGCCAAGG		
Mcu	F: ACTCACCAGATGGCGTTCG	129	NM-001033259
	R: CATGGCTTAGGAGGTCTCTCTT		
Sod1	F: AACCAGTTGTGTTGTCAGGAC	139	NM-011434
	R: CCACCATGTTTCTTAGAGTGAGG		
Ndufs3	F: TGGCAGCACGTAAGAAGGG	123	NM-026688
	R: CTTGGGTAAGATTTCAGCCACAT		
Sdha	F: GGAACACTCCAAAAACAGACCT	106	NM-023281
	R: CCACCACTGGGTATTGAGTAGAA		
Rela	F: TGCGATTCCGCTATAAATGCG	111	NM_009045
	R: ACAAGTTCATGTGGATGAGGC		

Slc9a1	F: CATCCTTGTCTTCGGGGAGTC	193	NM_016981
	R: GGAGGTGAAAGCTGCGATTAC		
Tiam1	F: GAAGCACACTTCACGCTCC	154	NM_009384
	R: CTCCAGGCCATTTTCAGCCA		
Pik3cd	F: TTATTGCGTGTCAGCAACCGA	215	NM_001164052
	R: TTGCCGTAGAGGTATTCGTGC		
Map2k2	F: GTTACCGGCACTCACTATCAAC	176	NM_023138
	R: CCTCCAGCCGCTTCCTTTG		
β -Actin	F: GGCTGTATTCCCCTCCATCG	154	NM-007393
	R: CCAGTTGGTAACAATGCCATGT		
